# A Review of Traumatic Axonal Injury following Whiplash Injury As Demonstrated by Diffusion Tensor Tractography

**DOI:** 10.3389/fneur.2018.00057

**Published:** 2018-02-08

**Authors:** Sung Ho Jang, Young Hyeon Kwon

**Affiliations:** ^1^Department of Physical Medicine and Rehabilitation, College of Medicine, Yeungnam University, Daegu, South Korea

**Keywords:** whiplash injury, diffusion tensor imaging, diffusion tensor tractography, mild traumatic brain injury, traumatic axonal injury, concussion

## Abstract

Whiplash is a bony or soft tissue injury resulting from an acceleration–deceleration energy transfer in the neck. Although patients with whiplash injury often complain of cerebral symptoms, and previous studies have reported evidence indicating brain injury, such an association has not been clearly elucidated. Traumatic axonal injury (TAI) is tearing of axons due to indirect shearing forces during acceleration, deceleration, and rotation of the brain or to direct head trauma. Diffusion tensor imaging (DTI) has a unique advantage to detect TAI in patients whose conventional brain CT or magnetic resonance imaging (MRI) results were negative following head trauma. Since the introduction of DTI, six studies using diffusion tensor tractography (DTT) based on DTI data have reported TAI in patients with whiplash injury, even though conventional brain CT or MRI results were negative. A precise TAI diagnosis in whiplash patients is clinically important for proper management and prognosis. Among the methods employed to diagnose TAI in the six previous studies, the common diagnostic approach for neural tract TAI in individual patients with whiplash injury were (1) whiplash injury history due to car accident; (2) development of new clinical symptoms and signs after whiplash injury; (3) evidence of neural tract TAI in DTT results, mainly *via* configurational analysis; and (4) coincidence of newly developed clinical manifestations and the function of injured neural tracts. All six studies were individual patient case studies; therefore, further prospective studies involving larger number of subjects should be encouraged.

## Introduction

Whiplash is a bony or soft tissue injury resulting from an acceleration–deceleration mechanism of energy transfer to the neck (1). Patients with whiplash injury often complain of cerebral symptoms suggestive of brain injury, such as headache, dizziness, sleeping problems, cognitive dysfunction, visual symptoms, and central pain (2, 3). Previous studies have reported the following evidences, which indicate brain injury in patients with whiplash injury: changes of blood flow or perfusion in functional neuroimaging studies, decreased gray matter density in voxel-based morphometry study, and hypoperfusion in single photon emission computed tomography and positron emission tomography (4–8).

Neural axons in the brain are reported to be vulnerable to mechanical loading through diffuse head trauma (9, 10). Traumatic axonal injury (TAI) is defined as the tearing of axons due to indirect shearing forces during acceleration, deceleration, and rotation of the brain or due to direct head trauma (11–17). A previous animal study reported that histopathology of TAI was demonstrated by acceleration–deceleration force in primates, especially in primates with prolonged coma (longer than 15 min) (18). In addition, they found that TAI was more severe when forces were produced in the coronal (lateral) plane than sagittal (flexion–extension) plane (18). Because conventional brain magnetic resonance imaging (MRI) is not sufficiently sensitive for detection of TAI, a diagnosis of TAI in live patients with whiplash was impossible for a long time (17, 19, 20). In the 1990s, following the introduction of diffusion tensor imaging (DTI), several studies used diffusion tensor tractography (DTT) results, which are derived from DTI data, to report on TAI in patients with whiplash whose conventional brain CT or MRI results were negative (21–26).

In this study, DTI studies that have demonstrated TAI in patients with whiplash are reviewed. Relevant studies reported between 1966 and 2017 were identified by accessing electronic databases (PubMed, Google Scholar, and MEDLINE). In those database searches, the following keywords were used: DTI, DTT, whiplash injury, brain injury, cerebral concussion, traumatic brain injury (TBI), TAI, and head trauma. This review is limited to studies of humans with whiplash injury. We excluded patients whose head had hit the car steering wheel or car windows to rule out the possibility of TAI due to direct head trauma. Finally, six studies that demonstrated TAI by performing DTT were selected for review and are discussed below (21–26).

### Usefulness of DTT in Detecting TAI in Patients with Whiplash Injury

The introduction of DTI began a new era in the diagnosis of subcortical white matter pathology in the live human brain, because DTI can provide invaluable information about subcortical white matter that cannot be obtained *via* conventional MRI (27). Initially, DTI was used to detect white matter pathologies undetectable by conventional CT or MRI in various brain pathologies including cerebral palsy, hypoxic-ischemic brain injury, and congenital brain disease (17). Since Arfanakis’s study in 2002, TAI has been demonstrated in hundreds of DTI studies of patients with TBI (12–14, 17, 19, 28). Among these studies, only six have demonstrated TAI of the neural tracts following whiplash injury due to car accidents (21–26).

**Figure 1:**
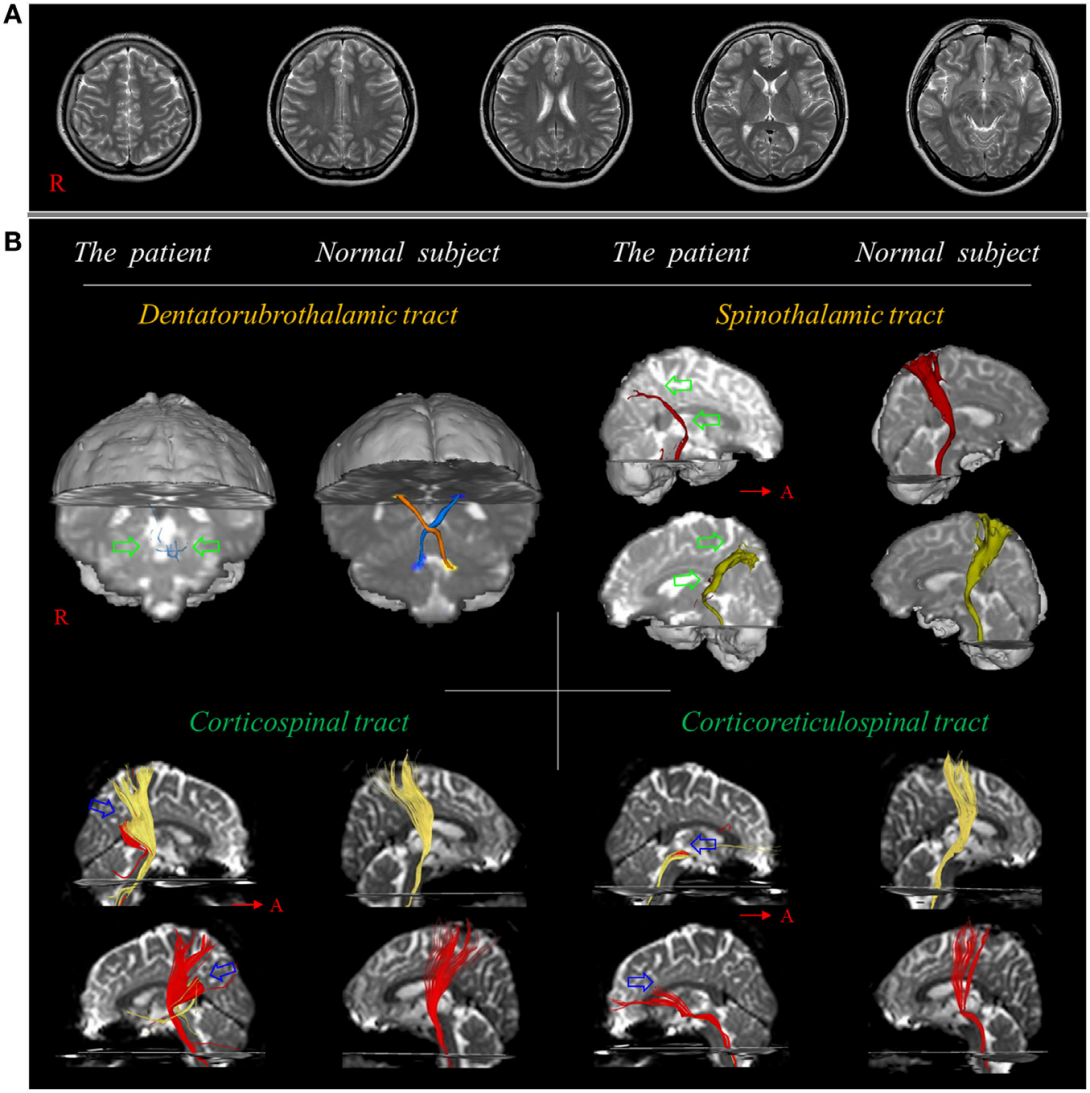
**(A)** T2-weighted brain MR images at 10 weeks after onset show no abnormality. **(B)** Results of diffusion tensor tractography. Only tiny fibers (green arrows) of the dentato-rubro-thalamic tract are reconstructed at the brainstem level and the spinothalamic tract is thinner in both hemispheres (green arrows) compared with a normal subject (26-year-old female). By contrast, the corticospinal tract and corticoreticulospinal tract show partial tearing and discontinuation at the subcortical white matter level in both hemispheres (blue arrows). Reprinted from Jang and Lee (26) with permission.

Two methods have been used to detect TAI: (1) region of interest (ROI) method in which measurement of DTI parameters in an ROI of the brain can be used for diagnosis of TAI and (2) DTT method involving analysis of DTT images of neural tracts. DTT allows for three-dimensional visualization and estimation of the neural tracts by permitting reconstruction of the neural tracts from DTI data; thus, TAI can be diagnosed by measurement of DTT parameters and/or from configurational analysis of the reconstructed neural tracts (12–14, 17, 19, 28). The ROI method can yield false results due to high inter-analyzer variability when establishing the ROI in the brain (29). In addition, ROI-based results can differ depending on whether the ROI is placed in a TAI lesion or in a normal-appearing area because a TAI lesion can exhibit configurational characteristics with partial tearing, narrowing, or discontinuation (17, 26). By contrast, DTT for reconstruction of the neural tracts usually employs an ROI method that reconstructs only those neural fibers passing through more than two ROIs (17). Because the area of the ROI and the reconstruction conditions for neural tracts are well defined for each neural tract, high repeatability and reliability of DTT neural tract results have been demonstrated (30–32). The main advantage of DTT over DTI is that in DTT the entire neural tract can be evaluated by examining several DTT parameters (i.e., fractional anisotropy; the degree of directionality of microstructures such as axons, mean diffusivity, the magnitude of water diffusion, and fiber number; and the number of voxels contained within a neural tract) and/or by undertaking configurational analysis (17, 27). Therefore, significant changes in DTT parameters and/or abnormal configurational analysis results in DTT (i.e., partial tearing, narrowing, or discontinuation) indicate injury of a neural tract (Figure 1) (12, 13, 17, 21–26). Although DTT is a powerful anatomic imaging tool that can demonstrate gross fiber architecture, it can also produce false-positive and false-negative results due to crossed fibers or a partial volume effect (29, 33).

### DTT Studies on TAI in Patients with Whiplash Injury

After the introduction of DTI, six studies using DTT have reported diagnoses of TAI of the corticospinal tract (CST), corticoreticulospinal tract (CRT), dentato-rubro-thalamic tract, ascending reticular activating system (ARAS), and spinothalamic tract (STT) in six patients with whiplash injury (Table 1) (21–26).

**Table 1:** DTT studies on traumatic axonal injury in patients with whiplash injury.

Reference	Publication year	Patient no.	Duration to DTT	Clinical features	Involved neural tracts	Diagnosis method on DTT
Kwon and Jang (21)	2014	1	10 weeks	Proximal weakness and gait disturbance	CRT	Configuration (discontinuation)

Seo and Jang (22)	2015	1	15 months	Fine motor impairment of hands	CST	Configuration (partial tearing)

Jang and Kwon (23)	2015	1	1 month	Tremor and ataxia	DRTT	Configuration (thinning)

Jang et al. (24)	2016	1	3 months	Ataxia	ICP	Configuration (discontinuation)
						DTT parameters (decreased fiber number)

Jang and Kwon (25)	2017	1	10 weeks	Excessive daytime sleepiness	ARAS	Configuration (thinning and partial tearing)
			10 months			

Jang and Lee (26)	2017	1	10 weeks	Weakness, tremor, ataxia, andcentral pain	DRTT	Configuration (thinning: DRTT and STT; partial tearing and discontinuation: CST and CRT)
					STT	
					CST	
					CRT	

*DTT, diffusion tensor tractography; CRT, corticoreticulospinal tract; CST, corticospinal tract; DRTT, dentato-rubro-thalamic tract; ICP, inferior cerebellar peduncle; ARAS, ascending reticular activating system; STT, spinothalamic tract*.

In 2014, Kwon and Jang reported a patient who, following whiplash injury, showed delayed gait disturbance due to injury of the CRT (21). The 14-year-old female patient suffered from injury in a car accident. While she was half-asleep in the backseat of a sedan that was stopped, the sedan was hit by another sedan from behind. During the accident, she hit her head on the backseat during hyperextension following flexion. She did not show loss of consciousness (LOC) or post-traumatic amnesia (PTA), and her Glasgow Coma Scale (GCS) score was 15 when she arrived at the hospital (34). She showed mild quadriparesis after onset; however, she began to show gait disturbance and aggravated quadriparesis with increasingly severe weakness of the proximal joints beginning 29 days after onset. Conventional brain MRI and electromyography study performed at 10 weeks after onset did not reveal any abnormality. However, discontinuation of the CRT at the midbrain level was observed in both hemispheres on DTT obtained at 10 weeks after onset. The authors concluded that the proximal weakness and gait disturbance in this patient appeared to be ascribed to TAI of both CRTs following the whiplash injury, and assumed that the aggravated weakness that started at 29 days after onset could be ascribed to secondary TAI (15, 16, 21).

In 2015, Seo and Jang reported on a patient who revealed TAI of the CST following whiplash injury (22). While driving his sedan, the 39-year-old male’s car collided with a truck; consequently, he experienced a whiplash injury during flexion–extension of his head. His TBI spectrum was compatible with mild TBI (LOC[−], PTA[−], and GCS[15]) (34). He experienced mild weakness in all four extremities and impairment of fine motor activities in both hands. At the time of DTI (15 months after onset), grip strength of both hands was within the normal range; however, fine motor functioning of both hands was mildly deteriorated and below the normal range. On the 15-month DTT, partial tearing of the CST at the subcortical white matter was observed in both hemispheres. However, the DTT results (i.e., fractional anisotropy, mean diffusivity, and fiber number) for both CSTs were within the normal range of control subjects. The motor evoked potentials obtained from both hand muscles showed normal latencies and low amplitudes, which indicate total amounts of the CST fibers. As a result, they demonstrated a TAI of the CST based on configurational analysis of DTT results and presence of low-amplitude motor-evoked potentials (22).

In 2015, Jang and Kwon reported a TAI of the dentatorubrospinal tract, which connects between the dentate nucleus in the cerebellum and the contralateral thalamic ventrolateral nucleus and is involved in motor coordination, in a patient following whiplash injury (23). The 41-year-old female experienced head trauma resulting from flexion–hyperextension injury when her car, while stopping at an intersection, was hit from behind by a moving car. The patient experienced LOC and PTA for approximately 1 min (34), and her GCS score was 15. No specific lesion was observed on brain MRI. However, 2 weeks after onset, the patient began to exhibit resting and intentional tremors and an ataxic gait. Her symptoms had become aggravated by the passage of time. On a 1-month DTT, the left dentatorubrospinal tract showed thinning compared with the right dentatorubrospinal tract. As a result, TAI of the dentatorubrospinal tract was diagnosed in a patient with tremor and ataxia following whiplash injury (23).

In 2016, Jang et al. reported on a patient with whiplash injury who showed TAI of the inferior cerebellar peduncle (ICP), which is involved in the control of balance by integrating proprioceptive and vestibular functions (24). The patient, a 42-year-old male, experienced head trauma from a whiplash (flexion–hyperextension) injury during a collision in which another sedan hit the patient’s sedan while he was stopping at an intersection. The patient lost consciousness for 10 s without PTA (34), and his GCS was 15 when he arrived at the hospital. Following the whiplash injury, the patient showed trunk ataxia and a balance problem during gait. No abnormal lesion was detected on conventional brain MRI, which was performed 3 months after onset. However, on a 3-month DTT, the right ICP was observed to be discontinued at the proximal portion of the transverse cerebellar branch of the ICP. In addition, the fiber number of the right ICP was lower, by more than 2 SDs, than that in control subjects. Consequently, based on DTT results, TAI of the ICP was demonstrated in a patient with balance problem following whiplash injury (24).

In 2017, Jang and Kwon reported on a patient who revealed aggravation of excessive daytime sleepiness (EDS) concurrent with aggravation of an injured ARAS following whiplash injury (25). The 42-year-old male suffered head trauma due to flexion–hyperextension injury after a collision in which his sedan was hit from behind by another sedan while he was stopping at an intersection. The patient lost consciousness for approximately 10 s but did not experience PTA (34). The patient’s GCS score was 15. Conventional brain MRI performed 10 weeks after onset did not show any abnormality. The patient complained of EDS after the head trauma, and there was aggravation of EDS with the passage of time. On 10-week DTT, decreased neural connectivity of the upper ARAS from intralaminar thalamic nuclei to the prefrontal cortex and basal forebrain was detected in both hemispheres. However, no significant abnormality was observed in the dorsal and ventral lower ARAS. In addition, on 16-month DTT, the left dorsal lower ARAS showed partial tearing and the ventral lower ARAS revealed thinning on both sides and partial tearing on the right side. The authors concluded that the aggravation of the lower dorsal and ventral ARAS appeared to be related to the aggravation of EDS in this patient (25).

In 2017, Jang and Lee reported on a patient who revealed severe and extensive TAI in several neural tracts following whiplash injury (26). The 26-year-old female experienced a flexion–hyperextension injury after being hit from behind by a slowly moving car. At the time of whiplash injury, she did not experience LOC or PTA, and her GCS score was 15 when she arrived at the hospital (34). She began to experience tremor in the right leg, and, from 5 days after onset, tremor also developed in the left leg. At 8 days after onset, she began to feel a tingling sensation in both legs. Conventional brain MRI at 2 weeks after onset did not show any abnormality. When she started rehabilitation at 10 weeks after onset, she exhibited mild quadriparesis with severe weakness of the proximal joint (shoulder/hip), along with severe resting and intentional tremors, ataxic gait, and severe myoclonus. Severe and extensive TAIs in several neural tracts (e.g., narrowing on both sides in the dentatorubrospinal tract and STT, and partial tearing and discontinuation at the subcortical white matter level in both sides of the CST and CRT) relevant to the patient’s clinical features were observed in this patient (Figure 1) (26).

## Conclusion

In this review, six DTT-based studies on TAI of the neural tracts in six patients with whiplash injury were reviewed. A precise diagnosis of TAI in patients with whiplash injury is clinically important for proper management and prognosis. Among the methods these six studies used to diagnose TAI of the neural tracts, the commonest diagnostic approaches for neural tract TAI in individual whiplash patients were (1) whiplash injury history due to car accident; (2) development of new clinical symptoms and signs subsequent to a whiplash injury; (3) evidence of neural tract TAI in DTT results, mainly from configurational analysis; and (4) coincidence of the newly developed clinical manifestations and the function of the injured neural tracts. However, we could not determine the vulnerable neural tracts by whiplash injury because all six studies were case reports on six individuals, which were focused on limited neural tracts relevant to the clinical features of each patient; therefore, further prospective studies involving a larger number of subjects is needed. Especially, analysis of many neural tracts or whole brain using tract-based spatial-statistics would be necessary to find the vulnerable neural tracts or brain regions by whiplash injury. On the other hand, four of the six patients showed delayed onset or aggravation of clinical features with the passage of time, which suggests the possibility of secondary TAI that refers to a condition in which axons were not injured at the time of head trauma, but axonal injury is caused by the sequential process of impaired axoplasmic transport, continued axonal swelling, and subsequent disconnection rather than primary TAI, which indicates that the axons are damaged by shear/strain injury at the time of head trauma (15, 16, 21, 23, 25, 26). Five of the six patients suffered the whiplash injury through a rear-end automobile–automobile collision (21, 23–26). Likewise, further prospective and follow-up studies should be warranted to clarify pathophysiology and injury mechanisms of TAI following whiplash injury.

## Author Contributions

SJ is supervisor. YK wrote the manuscript.

## Conflict of Interest Statement

Financial disclosure statements have been obtained, and no conflicts of interest have been reported by the authors or by any individuals in control of the content of this article.
